# Modulation of endothelial cell integrity and inflammatory activation by commercial lipid emulsions

**DOI:** 10.1186/s12944-015-0005-6

**Published:** 2015-02-18

**Authors:** Kevin A Harvey, Zhidong Xu, Thomas M Pavlina, Gary P Zaloga, Rafat A Siddiqui

**Affiliations:** Cellular Biochemistry Laboratory, Methodist Research Institute, Indiana University Health, 1800 N. Capitol Ave, E504D, Indianapolis, IN 46202 USA; Department of Medicine, Indiana University School of Medicine, Indianapolis, IN 46202 USA; Baxter Healthcare Corporation, Deerfield, IL 60015 USA

**Keywords:** Parenteral nutrition, Lipid emulsions, Lipids, Endothelial cells, Inflammation, Apoptosis, Fish oil, Olive oil, Soybean oil, Fatty acids, Nutrition

## Abstract

**Background:**

Thrombosis and immune dysfunction are two important complications that result from the administration of parenteral nutrition. Endothelial cells within the vasculature are crucial components necessary for maintenance of normal coagulation and immune function.

**Methods:**

We compared the effects of three commercial lipid emulsions (LEs; Intralipid®, ClinOleic® [or Clinolipid®], and Omegaven®) differing in the levels of omega-6 polyunsaturated fatty acids, omega-3 polyunsaturated fatty acids, omega-9 monounsaturated fatty acids, and saturated fatty acids upon endothelial cell fatty acid composition using Gas chromatography, endothelial cell integrity by assessing measurement of apoptosis and necrosis using flow cytometry, endothelial cell inflammatory activation by assessing the induction of ICAM-1 by lipopolysaccharide [LPS]), and transcription factor activation (phosphorylation of NF-κB) using western blot analysis.

**Results:**

Gas chromatographic analysis confirmed cellular uptake of the fatty acids within the LEs; furthermore, these fatty acid changes reflected the composition of the oils and egg phosphatides used in the manufacturing of these emulsions. However, the kinetics of fatty acid uptake and processing differed between LEs. Fish oil LE negatively impacted cell viability by doubling the percentage of apoptotic and necrotic cell populations quantified by flow cytometry using Annexin V/Fluorescein and propidium iodide. The soybean oil LE did not alter cell viability, while the olive oil-predominate emulsion improved cell viability. All LEs were capable of suppressing LPS-induced ICAM-1 expression; however, the fish oil LE was more potent than the other emulsions. Fish oil LE supplementation of cells also suppressed LPS-induced phosphorylation of NF-κB, while the soybean oil and olive predominant LE had no effect upon NF-κB phosphorylation.

**Conclusions:**

Lipid emulsions are readily incorporated and stored in the form of triacylglycerols. Soybean oil-based, olive oil-predominant and fish-oil based LEs differentially affected endothelial cell integrity. Importantly, these three LEs were capable of suppressing endothelial cell inflammatory response despite their fatty acid content.

## Background

Parenteral nutrition is an important therapeutic modality for the maintenance of nutritional status in patients with dysfunctional gastrointestinal tracts. Lipid is one component of the optimal nutritional intake of these patients and supplies both needed energy and essential fatty acids to the patients. Two important complications from the administration of parenteral nutrition are thrombosis and immune dysfunction [1,2]. The vascular endothelial cell is instrumental in the pathogenicity of both these complications [3]. Vascular endothelium plays a key role in hemostasis, regulation of vessel tone and tissue blood flow, oxygen and nutrient transport into the tissues, vascular permeability, and inflammation [3,4].

Vascular endothelial integrity is important for the maintenance of anti-coagulation factors on the surface of blood vessels and minimization of coagulation activation. Endothelial cell apoptosis is implicated in the pathogenicity of thrombosis [5]. Free fatty acids have multiple effects upon endothelial cells that include induction of apoptosis and /or necrosis [5-9]. However, the effects of complex fatty acid formulations such as commercial lipid emulsions (LEs), which are based upon mixtures of soybean, olive, coconut, and fish oils upon endothelial cell integrity (apoptosis and necrosis), have not previously been examined.

Endothelial cells also form an important component of the inflammatory and immune response systems and are one of the first cells to be activated during inflammation. Endothelial cell activation promotes leukocyte binding and transmigration at sites of inflammation via induction of adhesion molecules such as intercellular adhesion molecule-1 (ICAM-1) [10-12]. Inflammation-induced ICAM-1 expression has been linked to activation of the transcription factor, nuclear factor kappa B (NF-κB), by various inflammatory stimuli (i.e., lipopolysaccharide [LPS], tumor necrosis factor-alpha [TNFα], and interleukin-1 beta [IL-1β]). Most studies of vascular endothelial cell inflammatory activation have been performed using cultured endothelial cells with purified individual free fatty acids [8,9,13-15]. However, the studies using individual free fatty acids may not reflect the performance of complex LEs that contain mixtures of various fatty acids.

The objective of this study was to expand our knowledge of the effects of LEs, as opposed to free fatty acids, upon endothelial cell functions. We were particularly interested in those endothelial cell functions that related to thrombosis and inflammatory activation. In addition, we were interested in evaluating the effects of the LEs upon potential endothelial cell signaling pathways involved in thrombosis and inflammation. We compared the effects of three common commercial LEs differing in the levels of omega-6 polyunsaturated fatty acids (n-6 PUFA), omega-3 polyunsaturated fatty acids (n-3 PUFA), omega-9 monounsaturated fatty acids (n-9 MUFA), and saturated fatty acids upon endothelial cell fatty acid uptake and composition, endothelial cell integrity (assessed by measurement of apoptosis and necrosis), endothelial cell inflammatory activation (assessed through the induction of ICAM-1 by LPS), and transcription factor activation (phosphorylation of NF-κB). This is the first study to directly compare the three major classes of commercial LEs (i.e., soybean oil-based LE, olive oil-based LE, fish oil-based LE) upon these endothelial cell parameters and is the first study to evaluate the effects of LEs upon endothelial cell apoptosis/necrosis and NF-κB activation via phosphorylation. In addition to the varying fatty acid content present in the emulsions, these commercially available products also differ in tocopherol, tocotrienol, and sterol composition [16-18]. Our pre-study hypothesis was that the n-6 PUFA predominant LE (soybean oil) would enhance the endothelial NF-κB and ICAM-1 response to LPS (a pro-inflammatory response), the n-3 PUFA predominant LE (fish oil) would suppress the NF-κB and ICAM-1 response (an anti-inflammatory response), and the n-9 MUFA predominant LE (i.e., olive oil) would have a neutral effect on endothelial cell NF-κB activation and ICAM-1 response.

## Methods

### Materials

Human-derived aortic endothelial cells (HAECs), endothelial basal medium-2 (EBM-2), and the endothelial growth medium-2 microvascular (EGM-2MV) bullet kit materials were purchased from Lonza Incorporated (Walkersville, MD). Fetal bovine serum (FBS) and all electrophoresis products were purchased from Life Technologies (Grand Island, NY). Reagents, chemicals, and the Oil Red O staining kits were acquired from Sigma Chemical Company (St. Louis, MO). Consumable tissue culture products were obtained from Fisher Scientific (Pittsburgh, PA). The Annexin V FLUOS staining kits were purchased from Roche Applied Science (Indianapolis, IN). Gas chromatography standards were acquired from Restek Corporation (Bellefonte, PA). Fluorescently coupled antibodies were obtained from BD Pharmingen (San Diego, CA). Western blot antibodies were purchased from Cell Signaling Technology (Danver, MA).

### Human aortic endothelial cell culture

Human aortic endothelial cells (HAECs) were maintained in EMB-2 supplemented with bullet kit materials and 5% FBS. Cells were maintained at 37°C in 5% CO_2_ in a humidified atmosphere. Endothelial cell cultures in this study were utilized at 80-90% confluence and maintained for less than 10 passages.

### Lipid emulsions

A soybean oil-based LE that was enriched with omega-6 PUFA (SO; Intralipid®, Fresenius Kabi, Bad Homberg, Germany), an 80% olive plus 20% soybean LE enriched with omega-9 MUFA (OO; Clinolipid® or ClinOleic®, Baxter Healthcare, Deerfield, IL), and a fish oil-based LE enriched in omega-3 PUFA (FO; Omegaven®, Fresenius Kabi, Bad Homburg, Germany) were evaluated in the study (See Table 1). SO and OO were available as 20% LE (containing 20 g lipid/dl), while FO was available as a 10% LE.

**Table 1:** **Fatty acid composition of lipid emulsions (wt % composition)**

**FA common name/chemical name**		**OO**	**SO**	**FO**
**6:0**	Caproic/Hexanoic acid	0.06 ± 0.002	0.06 ± 0.002	0.14 ± 0.002
**8:0**	Caprylic/Octanoic acid	N/D	N/D	N/D
**10:0**	Capric/Decanoic acid	N/D	N/D	N/D
**12:0**	Lauric/dodecanoic acid	N/D	N/D	0.05 ± 0.002
**14:0**	Myristic/Tetradecanoic acid	0.01 ± 0.00	0.05 ± 0.002	4.69 ± 0.05
**15:0**	Pentadecylic/Pentadecanoic acid	N/D	N/D	0.36 ± 0.004
**16:0**	Palmitic/Hexadecanoic acid	13.04 ± 0.14	11.00 ± 0.16	11.62 ± 0.10
**16:1n-7**	Palmitoleic/Hexadecaenoic acid	0.91 ± 0.013	0.09 ± 0.002	8.05 ± 0.08
**16:2n-4**	Palmitdienoic/Hexadecadienoic acid	N/D	N/D	0.94 ± 0.01
**17:0**	Margaric/Heptadecanoic acid	0.04 ± 0.001	0.07 ± 0.002	0.24 ± 0.01
**16:3n-4**	Palmittrienoic/hexadecatrienoic acid	N/D	N/D	0.98 ± 0.01
**17:1n-7**	Heptadecaenoic	0.07 ± 0.001	0.04 ± 0.002	0.41 ± 0.01
**16:4n-1**	Palmitotetraenoic/Hexadecatetraenoic acid	N/D	0.02 ± 0.002	1.9 ± 0.02
**18:0**	Stearic/Octadecanoic acid	3.30 ± 0.04	3.90 ± 0.06	2.30 ± 0.02
**18:1n-9**	Oleic/Octadecaenoic acid	59.69 ± 0.72	20.92 ± 0.30	10.15 ± 0.09
**18:1n-7**	Vaccenic/Octadecaenoic acid	1.74 ± 0.11	1.29 ± 0.03	2.72 ± 0.02
**18:2n-6**	Linoleic/Octadecadienoic acid	18.56 ± 0.19	54.68 ± 0.79	2.98 ± 0.02
**18:3n-6**	γ-Linolenic (GLA)/Octadecatrienoic acid	N/D	N/D	0.25 ± 0.02
**18:3n-3**	α-Linolenic (ALA)/Octadecatrienoic acid	1.71 ± 0.02	6.65 ± 0.09	1.23 ± 0.01
**18:4n-3**	Stearidonic/Octadecatetraenoic acid	N/D	0.01 ± 0.00	4.56 ± 0.03
**20:0**	Arachidic/Eicosanoic acid	0.31 ± 0.004	0.27 ± 0.004	0.05 ± 0.003
**20:1n-9**	Gondoic/Eicosaenocic acid	0.19 ± 0.003	0.15 ± 0.002	1.10 ± 0.01
**20:1n-11**	Gadoleic/Eicosaenoic acid	N/D	0.03 ± 0.001	0.13 ± 0.01
**20:1n-7**	Paullinic/Eicosaenoic acid	N/D	N/D	0.10 ± 0.001
**20:2n-6**	Eicosadienoic/Eicosdienoic acid	N/D	0.01 ± 0.00	0.13 ± 0.01
**20:3n-9**	Mead acid/Eicosatrienoic acid	N/D	N/D	0.08 ± 0.01
**20:4n-6**	Arachidonic (AA)/Eicosatetraenoic acid	0.16 ± 0.002	0.18 ± 0.003	1.47 ± 0.02
**20:4n-3**	Eicostetraenoic	N/D	N/D	0.95 ± 0.01
**20:5n-3**	Timnodonic/Eicosapentaenoic acid (EPA)	N/D	N/D	19.34 ± 0.20
**21:5n-3**	Heneicosapentaenoic	N/D	N/D	0.64 ± 0.01
**22:0**	Behenic/Docosanoic acid	0.12 ± 0.002	0.31 ± 0.003	0.01 ± 0.001
**22:1n-9**	Erucic/Docosaenoic acid	N/D	N/D	0.13 ± 0.01
**22:1n-11**	Cetoleic/Docosaenoic acid	N/D	N/D	1.01 ± 0.003
**22:4n-6**	Adrenic/Docosatetraenoic acid	N/D	N/D	0.11 ± 0.004
**22:5n-6**	Osbond/Docosapentaenoic acid (DPA)	0.03 ± 0.003	0.01 ± 0.00	0.40 ± 0.01
**22:5n-3**	Clupanodonic/Docosapentaenoic acid	N/D	N/D	1.86 ± 0.02
**24:0**	Lignoceric/Tetracosanoic acid	0.05 ± 0.00	0.11 ± 0.004	N/D
**22:6n-3**	Cervonic/Docosahexaenoic acid (DHA)	0.06 ± 0.001	0.11 ± 0.001	17.67 ± 0.17
**24:1n-9**	Nervonic/Tetracosaenoic acid	N/D	0.06 ± 0.08	1.28 ± 1.12
**T-SFA**		16.62 ± 0.3	15.63 ± 0.28	15.73 ± 0.27
**T-MUFA**		63.39 ± 1.17	22.77 ± 0.53	23.68 ± 0.53
**T-PUFA**		20.00 ± 0.40	61.67 ± 1.56	57.54 ± 1.42
**Omega-3**		2.09 ± 0.04	5.66 ± 0.14	54.07 ± 1.28
**Omega-6**		17.90 ± 0.36	56.01 ± 1.42	3.47 ± 0.14
**Total**		100.00 ± 1.24	100.00 ± 1.54	100.00 ± 2.12

ND = Not detected.

### Lipid emulsion (fatty acid) cellular incorporation

Endothelial cells were supplemented with varying amounts (from 0.1% to 10%) of SO, OO, or FO for 24 hours under standard tissue culture conditions (see description above). Following treatments, cells were trypsinized and washed twice in calcium- and magnesium-free phosphate buffered saline (PBS) containing 0.1% fatty acid-free bovine serum albumin (BSA). To determine the total cellular fatty acid profile, an internal standard (C23:0) was added to a known volume of cell lysate, while protein content was calculated with the remaining sample using the bicinchoninic acid (BCA) protein assay kit (R & D Systems, Elysian, MN). Lipids were extracted using the Folch method, which requires the use of chloroform:methanol at a 2:1 ratio [19]. Fatty acid extracts from cell lysates were fractionated into phospholipid and triglyceride fractions following the addition of internal standards for each lipid class (C23:0). These lipid classes were fractionated with thin layer chromatography (Silica G, 20 x 20, 1000 μm; Analtech, Newark, DE) using a hexane:diethyl ether:acetic acid (70:30:1; by volume) solvent system. Phospholipid and triglyceride bands were collected and subjected to acid-catalyzed esterification by heating the samples for 90 minutes at 100°C while in a boron trifluoride-methanol solution (14%). The methyl ester form of the fatty acids were separated by gas chromatography (Shimadzu GC2010; Shimadzu, Columbia, MD) as previously described [20]. Fatty acid peaks were authenticated by comparing retention times to standards. Data were analyzed using Shimadzu’s GC Solutions software and normalized to protein content. Data were quantified as either the mean percentage of total identified fatty acids or as the mean quantity of each fatty acid.

### Assessment of lipid droplet formation

HAECs (1 x 10^4^) were plated in 4-well Permanox chamber slides and cultured overnight in complete media. LE was supplemented (1.0% v/v) for 24 hours under typical tissue culture conditions. In order to visualize the accumulation of the excess triglycerides, cells were stained for lipid droplets using an Oil Red O staining kit as described by the manufacturer. Modified Mayer’s hematoxylin was used as a counter-stain to visualize the cells. Images were obtained using an Olympus BX40 upright microscope, at 500x magnification with an oil immersion objective, and captured with Picture Frame imaging software.

### Apoptosis/necrosis detection

HAECs (1 x 10^5^) were grown in 6-well plates overnight in EBM-2 complete medium. Endothelial cells were supplemented with varying doses of LE (0.025-0.5%) in fresh medium for 24 hours under standard tissue culture conditions. Following the treatments, the spent medium containing any non-adherent cells was collected. The cells were rinsed in phosphate buffered saline (PBS), which was also collected, and the adherent cells were trypsinized and combined with the former material to ensure that both adherent and non-adherent cells were harvested for analysis. Cell pellets were washed once in PBS and resuspended into a labeling solution consisting of Annexin V FLUOS and propidium iodide. Cell suspensions were maintained at room temperature in the dark for 20 minutes prior to analysis on a FACSCalibur flow cytometer (Becton Dickinson, San Jose, CA), which was equipped with a 15 mW air-cooled argon-ion laser emitting at a 488 nm wavelength. Annexin V FLUOS was detected through a 530 nm band pass filter, while propidium iodide utilized a 630 nm long pass filter. Viable cells do not exhibit labeling by either fluorochrome; however, apoptotic cells bound the Annexin V FLUOS and necrotic cells were identified by the presence of both Annexin V and propidium iodide. Data were quantified using CellQuest software (Becton Dickinson, San Jose, CA) and represent the mean ± the standard deviation (SD) of three determinations.

### HAEC ICAM-1 membrane expression

Endothelial cells were grown in 6-well plates as described above. HAECs were supplemented with varying doses of LE (0.05-0.5%) for 24 hours prior to stimulation with lipopolysaccharide (LPS, 1 μg/mL) for 4 hours at 37°C to induce an inflammatory reaction. Following cell stimulation, HAECs were trypsinized, washed in PBS containing 0.5% BSA, and resuspended in 100 μL of PBS containing 0.5% BSA and 0.25 μg of phycoerythrin (PE)-conjugated intercellular adhesion molecule-1 (ICAM-1) antibody. ICAM-1 labeling occurred at room temperature in the dark for 20 minutes. Subsequently, the cells were washed in PBS supplemented with 0.5% BSA, and cell pellets were resuspended in 300 μL of the wash buffer and maintained at 4°C in the dark until analysis. An isotype antibody control was established for each data set to ensure binding specificity. Data analysis was performed on a FACSCalibur flow cytometer and CellQuest software as described above; however, the PE signal was detected through a 585 nm band pass filter. Results indicate the mean fluorescent intensity of gated endothelial cells, which exclude cellular debris.

### Western blot analysis for phosphorylated NF-kB

Subconfluent HAECs were grown in 6-well tissue culture-treated plates in the presence or absence of LE (0.05-0.5%) in EBM-2 complete medium for 24 hours under standard tissue culture conditions. HAECs were then stimulated with LPS (1 μg/mL) for 4 hours at 37°C to mimic an inflammatory insult. Treated cells were rinsed in cold PBS and lysed on ice for 15 minutes in a RIPA lysis buffer (10x; Millipore, Temecula, CA) containing 100 mM NaF, 2 mM Na_3_VO_4_, 2.5 mM diisopropyl fluorophosphate, and cOmplete mini protease cocktail inhibitor tablets (Roche Applied Science, Indianapolis, IN). A BCA protein assay kit was used to determine the protein content following sample centrifugation to remove the insoluble matter from the detergent solubilized extracts. Linearized proteins were electrophoretically separated in 4-12% polyacrylamide gradient gels and transferred onto nitrocellulose membranes. Membranes were blocked for 30 minutes at room temperature in 10% Roche western blocking reagent in Tris-buffered saline supplemented with 0.1% Triton X-100 (TBST). Blots were probed with primary antibodies in accordance with the manufacturer’s recommendations. Secondary antibodies were peroxidase-conjugated for protein detection using an enhanced chemiluminescence (ECL) system (Amersham Pharmacia Biotechnology, Piscataway, NJ). Nitrocellulose membranes were stripped in Restore western blot stripping buffer (Thermo Scientific, Rockford, IL) for 15 minutes at room temperature. Stripped blots were washed 6 times in TBST, blocked, and reprobed with an alternative antibody.

### Statistical analysis

Data represent the mean ± the standard deviation (SD) of at least three determinations. Student’s *t-*test was used to signify statistically significant differences between treatment groups and vehicle control. One-way ANOVA with Tukey’s post hoc test using SPSS Statistics 20 software was performed to test differences between groups. When a calculated *p* value of <0.05 is reported, statistical significance is indicated with an asterisk.

## Results

### Lipid emulsion cellular incorporation

**Figure 1:**
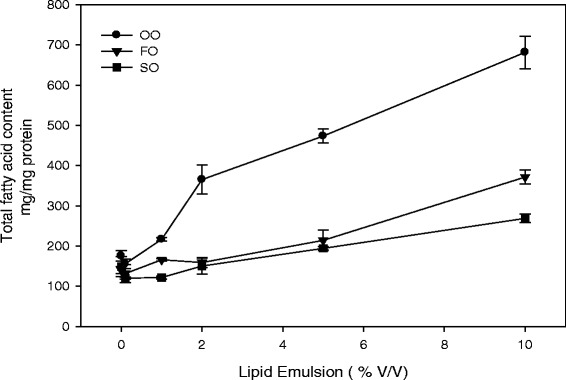
**Concentration of total fatty acids in endothelial cells following lipid emulsion supplementation.** Cells were supplemented with varying amounts of olive oil (OO)-, soybean oil (SO)-, or fish oil (FO)-based lipid emulsion. The amounts of total fatty acids were determined as the sum of all detectable fatty acids reported in Table 1. Each point represents the mean ± SD of three determinations.

HAECs were dose dependently supplemented with lipid emulsions (0.1-10%). Separate vehicle (PBS-supplemented) cells were used with each emulsion. Incorporation of total fatty acids in HAECs varied with different lipid emulsions, as shown in Figure 1. The total fatty acid uptake was lowest in SO-based LE, whereas it was highest in OO-based lipid emulsion (2–2.5 fold higher compared to SO). Supplementation with FO-based LE demonstrated an intermediate increase in total fatty acid uptake. The relative percentages of key identified fatty acids in total lipid extracts from the endothelial cells are presented in Tables 2, 3 and 4.

**Table 2:** **Fatty acid profile in OO-supplemented HAECs**

**Fatty acid**	**Vehicle**	**0.10%**	**1%**	**2%**	**5%**	**10%**
8:0	0.76 ± 0.21	0.24 ± 0.03*	0.10 ± 0.00*	0.12 ± 0.01*	0.12 ± 0.04*	0.06 ± 0.05*
10:0	0.52 ± 0.35	0.25 ± 0.00	0.06 ± 0.00	0.14 ± 0.03*	0.10 ± 0.02*	0.14 ± 0.05*
12:0	0.19 ± 0.10	0.19 ± 0.10	0.11 ± 0.02	0.07 ± 0.02	0.04 ± 0.00*	0.08 ± 0.05
14:0	3.51 ± 0.41	3.02 ± 0.27	1.03 ± 0.15*	0.71 ± 0.11*	0.60 ± 0.04*	0.53 ± 0.00*
16:0	26.37 ± 2.33	25.09 ± 1.81	27.39 ± 1.71	24.32 ± 2.25	25.09 ± 1.11	25.33 ± 1.39
16:1n-7	4.31 ± 0.01	3.05 ± 0.18*	1.39 ± 0.04*	1.51 ± 0.11*	1.45 ± 0.03*	1.39 ± 0.10*
18:0	16.50 ± 2.01	17.27 ± 1.98	10.89 ± 0.96	11.69 ± 1.24*	9.96 ± 0.14*	8.35 ± 0.40*
18:1n-9	20.94 ± 0.76	23.31 ± 2.13	30.97 ± 1.73*	33.23 ± 3.44*	32.27 ± 0.53*	31.15 ± 2.01*
18:1n-7	8.13 ± 0.03	7.59 ± 0.12*	2.74 ± 0.18*	3.90 ± 0.63*	3.07 ± 0.22*	2.64 ± 0.19*
18:2n-6	1.50 ± 0.16	2.12 ± 0.13	10.26 ± 0.66*	11.08 ± 0.86*	13.63 ± 0.59*	15.81 ± 0.91*
18:3n-6	0.16 ± 0.02	0.19 ± 0.05	1.30 ± 0.11*	0.50 ± 0.07*	0.73 ± 0.01*	0.73 ± 0.09*
18:3n-3	0.05 ± 0.01	0.05 ± 0.03	0.04 ± 0.01	0.19 ± 0.01*	0.25 ± 0.00*	0.35 ± 0.02*
20:0	0.17 ± 0.02	0.14 ± 0.03	0.05 ± 0.01	0.15 ± 0.00	0.13 ± 0.00*	0.11 ± 0.00*
20:4n-6	10.78 ± 0.18	11.29 ± 0.47	8.77 ± 0.46	7.83 ± 0.79*	8.01 ± 0.67*	8.90 ± 0.40*
20:5n-3	0.19 ± 0.00	0.20 ± 0.04	0.06 ± 0.00*	0.11 ± 0.02*	0.10 ± 0.02*	0.09 ± 0.01*
22:0	0.23 ± 0.02	0.19 ± 0.02	0.19 ± 0.06	0.12 ± 0.02*	0.08 ± 0.00*	0.05 ± 0.00*
22:5n-6	1.62 ± 0.02	1.75 ± 0.05	1.16 ± 0.05*	1.11 ± 0.06*	1.19 ± 0.11*	1.20 ± 0.07*
22:5n-3	1.57 ± 0.04	1.56 ± 0.16	0.55 ± 0.03*	0.67 ± 0.03*	0.52 ± 0.03*	0.45 ± 0.03*
24:0	0.47 ± 0.01	0.38 ± 0.04	0.07 ± 0.00	0.13 ± 0.01*	0.09 ± 0.00*	0.07 ± 0.00*
22:6n-3	2.03 ± 0.09	2.14 ± 0.00	2.88 ± 0.15*	2.42 ± 0.13*	2.57 ± 0.09*	2.57 ± 0.15*
SFA	48.72 ± 5.97	46.75 ± 4.29	39.89 ± 2.91*	37.45 ± 3.69*	36.21 ± 1.36*	34.74 ± 1.95*
MUFA	33.39 ± 0.80	33.95 ± 2.43	35.09 ± 1.95	38.64 ± 4.18	36.80 ± 0.77	35.17 ± 2.29
n-6 PUFA	14.06 ± 0.38	15.35 ± 0.69	20.23 ± 1.18*	20.52 ± 1.77*	23.56 ± 1.38*	26.64 ± 1.47*
n-3 PUFA	3.84 ± 0.14	3.95 ± 0.23	4.79 ± 0.29*	3.39 ± 0.18	3.44 ± 0.14	3.45 ± 0.21
Total	100 ± 7.28	100.00 ± 7.63	100.00 ± 6.33	100.00 ± 9.84	100.00 ± 3.66	100.00 ± 5.92

HAECs were supplemented with varying doses of OO for 24 hours under standard tissue culture conditions prior to analysis. Total lipids were extracted as described in the methods. Quantification was based on the use of an internal standard and normalization to protein content. Results are expressed as the mean wt% ± SD of three determinants. *Denotes statistically significant differences (p ≤ 0.05) compared to vehicle control.

**Table 3:** **Fatty acid profile in SO-supplemented HAECs**

**Fatty acid**	**Vehicle**	**0.10%**	**1%**	**2%**	**5%**	**10%**
8:0	0.41 ± 0.16	0.25 ± 0.07*	0.25 ± 0.05	0.28 ± 0.00	0.17 ± 0.02*	0.11 ± 0.00*
10:0	0.62 ± 0.28	0.43 ± 0.08	0.14 ± 0.01	0.48 ± 0.08	0.32 ± 0.01*	0.19 ± 0.05*
12:0	0.16 ± 0.05	0.19 ± 0.09	0.27 ± 0.04	0.24 ± 0.16	0.17 ± 0.01	0.11 ± 0.02
14:0	4.08 ± 0.85	3.75 ± 0.04	1.24 ± 0.06*	1.40 ± 0.08*	1.01 ± 0.04*	0.74 ± 0.10*
16:0	25.99 ± 4.34	25.07 ± 0.37	26.03 ± 1.38	24.05 ± 2.75	24.29 ± 0.32	24.30 ± 0.88
16:1n-7	4.16 ± 0.47	3.84 ± 0.04	1.05 ± 0.07*	1.96 ± 0.14*	1.55 ± 0.02*	1.49 ± 0.11*
18:0	16.94 ± 2.65	17.28 ± 0.25	13.89 ± 0.65	17.01 ± 1.76	15.34 ± 0.13	12.75 ± 0.69
18:1n-9	21.04 ± 3.08	20.60 ± 0.51	20.73 ± 1.11	20.64 ± 2.91	20.55 ± 0.22	20.13 ± 0.83
18:1n-7	8.34 ± 1.39	8.06 ± 0.18	2.43 ± 0.13*	4.23 ± 0.56*	3.43 ± 0.00*	2.59 ± 0.05*
18:2n-6	1.57 ± 0.24	2.08 ± 0.05	14.21 ± 0.85*	10.28 ± 1.97*	12.41 ± 0.75*	15.43 ± 0.52*
18:3n-6	0.16 ± 0.07	0.15 ± 0.01	0.82 ± 0.05*	0.44 ± 0.10*	0.68 ± 0.12*	1.12 ± 0.00*
18:3n-3	0.02 ± 0.01	0.01 ± 0.01	0.37 ± 0.03*	0.39 ± 0.09*	0.36 ± 0.00*	0.40 ± 0.02*
20:0	0.23 ± 0.04	0.20 ± 0.00	0.05 ± 0.01	0.25 ± 0.00	0.21 ± 0.01	0.17 ± 0.02
20:4n-6	10.22 ± 2.01	11.40 ± 0.25	10.81 ± 0.66	11.51 ± 1.36	11.96 ± 0.08	12.61 ± 0.37
20:5n-3	0.20 ± 0.04	0.20 ± 0.00	0.29 ± 0.03	0.16 ± 0.04	0.20 ± 0.02	0.24 ± 0.01
22:0	0.26 ± 0.05	0.22 ± 0.00	0.12 ± 0.01	0.22 ± 0.02	0.18 ± 0.01	0.14 ± 0.01*
22:5n-6	1.52 ± 0.38	1.78 ± 0.03	0.94 ± 0.04	1.19 ± 0.21	1.24 ± 0.02	1.22 ± 0.06
22:5n-3	1.62 ± 0.17	1.66 ± 0.09	1.06 ± 0.07	1.31 ± 0.24	1.10 ± 0.02*	0.84 ± 0.00*
24:0	0.43 ± 0.15	0.35 ± 0.02	0.12 ± 0.01	0.23 ± 0.06*	0.14 ± 0.02*	0.13 ± 0.01*
22:6n-3	1.91 ± 0.29	2.34 ± 0.06	5.18 ± 0.31*	4.05 ± 0.65*	4.98 ± 0.32*	5.68 ± 0.14*
SFA	49.11 ± 8.58	47.74 ± 0.94	42.11 ± 2.22	44.17 ± 4.91	41.85 ± 0.59	38.62 ± 1.77
MUFA	33.54 ± 4.93	32.50 ± 0.72	24.20 ± 1.31*	26.83 ± 3.61*	25.53 ± 0.25*	24.21 ± 0.99*
n-6 PUFA	13.46 ± 2.71	15.41 ± 0.33	26.33 ± 1.59*	23.42 ± 3.64*	26.30 ± 0.98*	30.38 ± 0.95*
n-3 PUFA	3.76 ± 0.50	4.22 ± 0.17	7.36 ± 0.45*	5.91 ± 1.02	6.63 ± 0.36	7.16 ± 0.18*
Total	99.87 ± 16.73	99.87 ± 2.17	100.00 ± 5.57	100.33 ± 13.18*	100.31 ± 2.18*	100.36 ± 3.89

HAECs were supplemented with varying doses of SO for 24 hours under standard tissue culture conditions prior to analysis. Total lipids were extracted as described in the methods. Quantification was based on the use of an internal standard and normalization to protein content. Results are expressed as the mean wt% ± SD of three determinants. *Denotes statistically significant differences (p ≤ 0.05) compared to vehicle control.

**Table 4:** **Fatty acid profile in FO-supplemented HAECs**

**Fatty acid**	**Vehicle**	**0.10%**	**1%**	**2%**	**5%**	**10%**
8:0	0.18 ± 0.04	0.25 ± 0.03	0.52 ± 0.11*	0.20 ± 0.03	0.20 ± 0.03	0.08 ± 0.03*
10:0	0.24 ± 0.09	0.26 ± 0.06	0.46 ± 0.05*	0.15 ± 0.03	0.18 ± 0.08	0.11 ± 0.05
12:0	0.18 ± 0.02	0.13 ± 0.00*	0.31 ± 0.06*	0.14 ± 0.01	0.14 ± 0.09	0.06 ± 0.02*
14:0	3.61 ± 0.18	4.27 ± 0.79	3.96 ± 1.06	5.35 ± 0.20*	1.47 ± 0.10*	1.05 ± 0.06*
16:0	26.56 ± 1.37	22.37 ± 3.35	21.85 ± 1.87*	22.85 ± 1.10*	19.26 ± 2.26*	20.23 ± 1.40*
16:1n-7	4.86 ± 0.31	5.69 ± 1.48	4.98 ± 1.74	7.28 ± 0.10*	2.36 ± 0.35*	1.84 ± 0.07*
18:0	16.90 ± 1.29	16.63 ± 2.07	15.39 ± 0.20	7.87 ± 0.48*	12.21 ± 1.18*	8.82 ± 0.31*
18:1n-9	21.97 ± 1.29	21.35 ± 3.63	25.59 ± 0.46*	25.58 ± 1.74	35.53 ± 4.81*	39.75 ± 1.89*
18:1n-7	8.47 ± 0.72	8.82 ± 1.62	4.78 ± 0.84*	4.25 ± 0.10*	3.44 ± 0.26*	2.81 ± 0.00*
18:2n-6	1.13 ± 0.13	2.63 ± 0.73	3.81 ± 0.26*	6.78 ± 0.46*	6.55 ± 0.89*	8.39 ± 0.45*
18:3n-6	0.13 ± 0.01	0.13 ± 0.01	0.25 ± 0.03*	0.27 ± 0.02*	0.30 ± 0.04*	0.31 ± 0.01*
18:3n-3	0.03 ± 0.02	0.17 ± 0.06*	0.26 ± 0.15	0.59 ± 0.02*	0.06 ± 0.04	0.13 ± 0.00*
20:0	0.26 ± 0.04	0.31 ± 0.04	0.42 ± 0.08*	0.20 ± 0.01	0.25 ± 0.02	0.13 ± 0.01*
20:4n-6	9.85 ± 0.53	9.37 ± 1.29	7.41 ± 0.30*	8.51 ± 0.39	8.31 ± 0.81	7.48 ± 0.27*
20:5n-3	0.21 ± 0.03	0.77 ± 0.24*	2.83 ± 0.59*	2.87 ± 0.16*	1.77 ± 0.09*	1.87 ± 0.08*
22:0	0.28 ± 0.01	0.27 ± 0.04	0.31 ± 0.00	0.12 ± 0.01*	0.16 ± 0.01*	0.07 ± 0.01*
22:5n-6	1.47 ± 0.07	1.29 ± 0.19	0.97 ± 0.03*	0.79 ± 0.07*	1.27 ± 0.11	1.14 ± 0.00*
22:5n-3	1.42 ± 0.05	2.46 ± 0.40*	1.46 ± 0.01	1.20 ± 0.12	1.78 ± 0.23	1.53 ± 0.03
24:0	0.35 ± 0.00	0.37 ± 0.02	0.47 ± 0.03*	0.11 ± 0.01*	0.20 ± 0.03*	0.07 ± 0.01*
22:6n-3	1.90 ± 0.06	2.47 ± 0.48	3.97 ± 0.20*	4.89 ± 0.29*	4.55 ± 0.61*	4.12 ± 0.02*
SFA	48.55 ± 3.05	44.85 ± 6.60	43.68 ± 3.46	36.98 ± 1.88*	34.08 ± 3.80*	30.63 ± 1.91*
MUFA	35.30 ± 2.32	35.86 ± 6.73	35.36 ± 3.04	37.11 ± 1.94	41.32 ± 5.41	44.41 ± 1.95*
n-6 PUFA	12.59 ± 0.74	13.42 ± 2.22	12.44 ± 0.61	16.27 ± 0.93*	16.43 ± 1.85*	17.32 ± 0.73*
n-3 PUFA	3.56 ± 0.16	5.86 ± 1.18*	8.52 ± 0.95*	9.63 ± 0.60*	8.16 ± 0.98*	7.64 ± 0.13*
Total	100.00 ± 6.27	100.00 ± 16.74	100.00 ± 8.07	100.00 ± 5.34	100.00 ± 12.04	100.00 ± 4.72

HAECs were supplemented with varying doses of FO for 24 hours under standard tissue culture conditions prior to analysis. Total lipids were extracted as described in the methods. Quantification was based on the use of an internal standard and normalization to protein content. Results are expressed as the mean wt% ± SD of three determinants. *Denotes statistically significant differences (p ≤ 0.05) compared to vehicle control.

In the vehicle (PBS-supplemented) cells, the saturated fatty acid class represented nearly one-half of all selected fatty acids (Tables 2, 3 and 4). MUFA were the second most abundant class of fatty acids, followed by n-6 PUFA and n-3 PUFA. As the percentage of OO supplementation increased (Table 2), the proportion of oleic and linoleic acid, and to a lesser extent α- and γ-linolenic acids and docosahexaenoic acid, increased in a dose-dependent manner. Palmitic and arachidonic acids maintained a consistent presence independent of lipid emulsion supplementation; furthermore, relative levels of myristic acid and the MUFAs, palmitoleic and vaccenic, declined. SO-supplemented endothelial cells (Table 3) demonstrated dose-dependent increases in the relative percentages of linoleic and γ-linolenic acids. The saturated fatty acids, with the exception of palmitic, displayed dose-dependent decreases. The percentage of oleic acid content was unchanged but levels of total MUFA were decreased. The FO-supplemented endothelial cells were administered the same volume/volume dose as OO and SO. However, the lipid concentration of FO was 10% compared to 20% in the OO and SO emulsions. Dose-dependent increases in the proportion of DHA and EPA were observed (Table 4) with the FO emulsion; however, the relative percentages of docosapentaenoic acid and α-linolenic acid did not substantially increase. The saturated fatty acid component decreased, whereas a significant increase in the percentage of oleic and linoleic acids was observed.

### Phospholipid and triglyceride fatty acid characterization

As shown in Figure 1, the overall cellular fatty acid content increased following LE supplementation in a dose-dependent manner. Thus, we determined whether the fatty acids were incorporated into cellular triglyceride (TG) and/or phospholipid (PL) fractions (Table 5). Minimal amounts of triglycerides were detected in non-supplemented endothelial cells, which resulted in many non-detectable fatty acids; however, significant levels were detected in all LE-supplemented cells (Table 5a). The fatty acid incorporation in TG mimicked the fatty acid profiles present in the lipid emulsions. However, the amount of fatty acid incorporated into TG and PL fractions varied with different LEs. In untreated HAECs, 95% of total fatty acids were present in the PL fraction and 5% in the TG fraction. Following OO-based LE supplementation, fatty acids were equally distributed in TG (51%) and PL (49%) fractions, whereas in SO-based LE supplementation, 36% and 64% of total fatty acids were present in the TG and PL fractions, respectively. In the FO-supplemented cells, more fatty acids were incorporated into the TG fraction (61%) with lesser amounts in the PL fraction (39%). The long-chain saturated fatty acids (>20 carbon chain length) were not detected in any of the triglyceride fractions.

**Table 5:** **Analysis of FA in triglyceride and phospholipid fractions**

	**(a) Triglyceride**					**(b) Phospholipid**			
**Fatty acid**	**Vehicle**	**OO**	**SO**	**FO**	**Fatty acid**	**Vehicle**	**OO**	**SO**	**FO**
C8:0	0.14 ± 0.05a	0.06 ± 0.00b	0.15 ± 0.08a	0.10 ± 0.04a	C8:0	0.13 ± 0.04a	0.04 ± 0.00b	0.11 ± 0.03a	0.16 ± 0.05a
C10:0	0.05 ± 0.01a	0.03 ± 0.01a	0.09 ± 0.01a	0.06 ± 0.03a	C10:0	0.07 ± 0.01a	0.03 ± 0.00a	0.05 ± 0.01a	0.10 ± 0.05a
C12:0	0.14 ± 0.02a	0.07 ± 0.02b	0.21 ± 0.08a	0.03 ± 0.01b	C12:0	0.15 ± 0.06a	0.04 ± 0.01b	0.06 ± 0.00b	0.05 ± 0.04b
C14:0	0.42 ± 0.04a	0.41 ± 0.05a	0.42 ± 0.02a	0.81 ± 0.07b	C14:0	3.36 ± 0.30a	0.62 ± 0.25b	0.82 ± 0.10b	0.92 ± 0.37b
C16:0	1.19 ± 0.42a	12.98 ± 1.67b	8.06 ± 0.92c	12.92 ± 1.74b	C16:0	25.93 ± 1.65a	14.41 ± 1.74b	17.97 ± 1.83b	11.15 ± 1.60b
C16:1n7	0.17 ± 0.06a	0.67 ± 0.07b	0.33 ± 0.03c	0.48 ± 0.08d	C16:1n7	3.69 ± 0.20a	0.72 ± 0.02b	0.72 ± 0.10b	0.43 ± 0.05c
C18:0	0.54 ± 0.15a	3.47 ± 0.42b	2.97 ± 0.30b	3.24 ± 0.39b	C18:0	15.79 ± 0.70a	7.43 ± 1.51b	10.93 ± 1.01b	5.99 ± 1.40b
C18:1n-9	0.85 ± 0.32a	19.41 ± 2.31b	8.50 ± 0.91c	22.12 ± 3.65b	C18:1n-9	20.82 ± 1.09a	11.56 ± 1.14b	12.22 ± 1.30b	10.26 ± 2.78b
C18:1 n-7	0.19 ± 0.07a	1.05 ± 0.24b	0.51 ± 0.06c	1.17 ± 0.17b	C18:1 n-7	8.16 ± 0.40a	1.69 ± 0.12b	1.92 ± 0.21b	1.21 ± 0.18b
C18:2n-6	0.70 ± 0.99a	6.07 ± 0.82b	7.54 ± 1.01b	7.53 ± 1.31b	C18:2n-6	1.39 ± 0.05a	4.19 ± 0.51b	6.66 ± 0.69c	2.98 ± 0.54b
C18:3n-3	N/D	0.98 ± 0.17a	0.51 ± 0.06b	0.17 ± 0.03c	C18:3n-3	0.15 ± 0.02a	0.32 ± 0.04b	0.31 ± 0.04b	0.11 ± 0.03a
C18:3n-6	N/D	N/D	0.29 ± 0.05a	0.08 ± 0.01b	C18:3n-6	0.05 ± 0.02a	0.04 ± 0.01a	0.08 ± 0.02a	0.03 ± 0.01a
C20:0	N/D	N/D	N/D	N/D	C20:0	0.10 ± 0.00a	0.05 ± 0.02b	0.05 ± 0.02b	0.06 ± 0.02b
C20:4n-6	N/D	4.02 ± 0.49a	3.80 ± 0.51a	4.96 ± 0.76a	C20:4n-6	9.74 ± 0.16a	4.75 ± 0.43b	7.00 ± 0.82c	3.54 ± 0.59b
C20:5n-3	N/D	N/D	0.15 ± 0.02a	2.45 ± 0.40b	C20:5n-3	0.12 ± 0.01a	0.06 ± 0.01b	0.14 ± 0.04a	0.66 ± 0.09c
C22:0	0.13 ± 0.02	N/D	N/D	N/D	C22:0	0.22 ± 0.03a	0.19 ± 0.13a	0.12 ± 0.02b	0.11 ± 0.02b
C22:5n-6	N/D	0.45 ± 0.07a	0.25 ± 0.01b	0.78 ± 0.15a	C22:5n-6	1.28 ± 0.03a	0.71 ± 0.04b	0.70 ± 0.08b	0.44 ± 0.06c
C22:5n-3	N/D	0.20 ± 0.03a	0.35 ± 0.05b	0.82 ± 0.32c	C22:5n-3	1.62 ± 0.04a	0.35 ± 0.02b	0.71 ± 0.09c	0.32 ± 0.06b
C24:0	N/D	N/D	N/D	N/D	C24:0	0.30 ± 0.03a	0.07 ± 0.01b	0.12 ± 0.01c	0.08 ± 0.01b
C22:6n-3	N/D	1.28 ± 0.17a	2.00 ± 0.31a,b	2.82 ± 0.54b,c	C22:6n-3	2.26 ± 0.07a	1.60 ± 0.14b	3.18 ± 0.31a	0.84 ± 0.24c
SFA	2.60 ± 0.72a	17.01 ± 2.17b	11.90 ± 1.41c	17.16 ± 2.27b	SFA	46.05 ± 2.82a	22.88 ± 3.66b	30.21 ± 3.04c	18.64 ± 3.57b
MUFA	1.21 ± 0.46a	21.13 ± 2.62b	9.34 ± 1.00c	23.77 ± 3.90b	MUFA	32.67 ± 1.69a	13.9 ± 61.28b	14.86 ± 1.61b	11.90 ± 3.01b
n-6 PUFA	0.70 ± 0.99a	10.55 ± 1.39b	11.88 ± 1.57b	13.35 ± 2.24b	n-6 PUFA	12.45 ± 0.25a	9.68 ± 0.98b	14.44 ± 1.60a	6.99 ± 1.20c
n-3 PUFA	N/D	2.45 ± 0.37a	3.01 ± 0.43a	6.27 ± 1.28b	n-3 PUFA	4.15 ± 0.15a	2.33 ± 0.20b	4.35 ± 0.47a	1.92 ± 0.42b
TOTAL	4.52 ± 2.18a	51.14 ± 6.54b	36.14 ± 4.42c	60.55 ± 9.69b	TOTAL	95.32 ± 4.91a	48.86 ± 6.12b	63.86 ± 6.72c	39.45 ± 8.20b

HAECs were supplemented with lipid emulsions (1%) for 24 hours under standard tissue culture conditions prior to analysis. Triglycerides (a) and Phospholipids (b) were resolved using thin layer chromatography and extracted as described in the methods. An “ND” indicates the lack of fatty acid detection. Results are expressed as the mean (wt%) ± SD of three experiments. One-way ANOVA with Tukey’s post hoc test using SPSS Statistics 20 software was performed to test differences between groups. The numbers with different letters represent a statistically significant difference p < 0.05.

**Figure 2:**
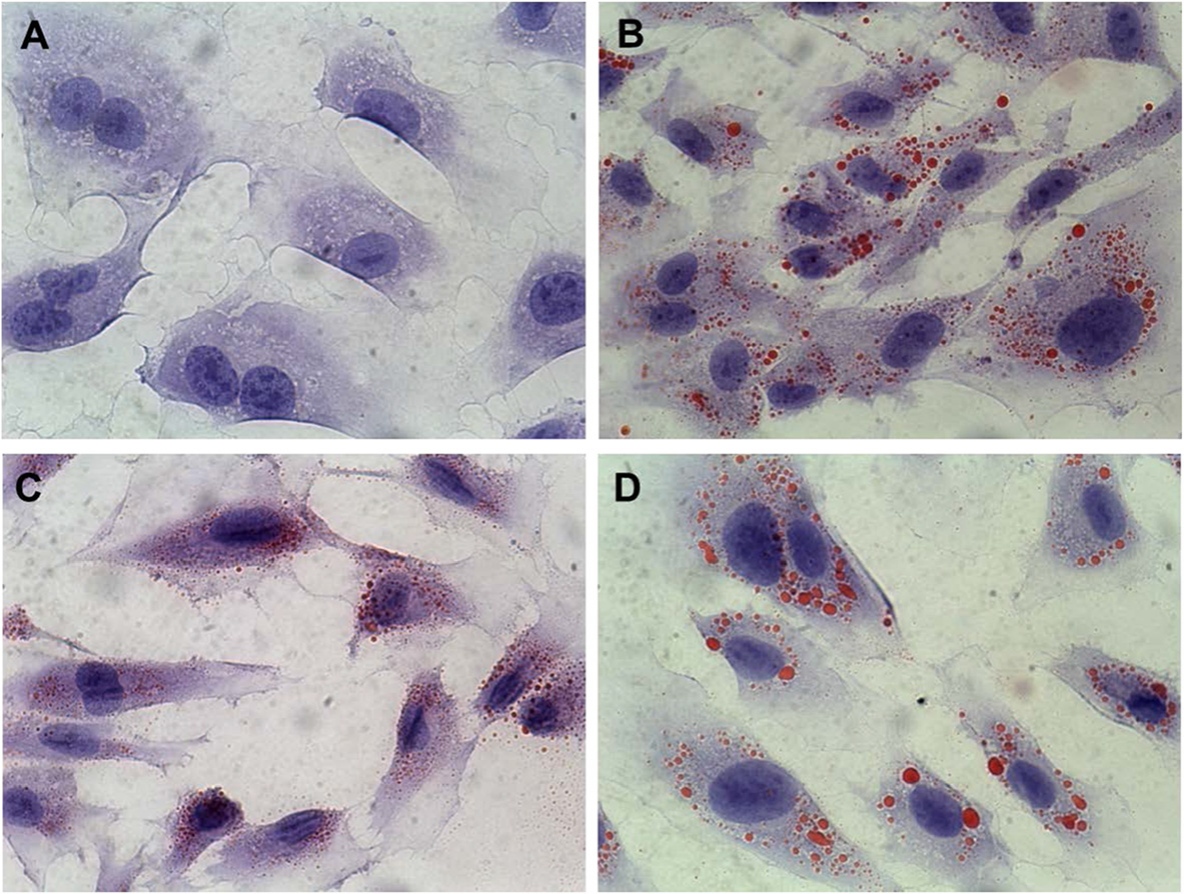
**Effect of lipid emulsion supplementation on the accumulation of neutral lipid storage.** Endothelial cells (PBS, **A**) were cultured for 24 hours in the presence of 1% (v/v) OO **(B)**, SO **(C)**, or FO **(D)**. Triglyceride-containing lipid droplets were stained with Oil Red O, while cells were counterstained with hematoxylin for visualization.

Oil Red O staining, which detects neutral cellular lipids in the form of lipid droplets, was used to confirm the triglyceride accumulation in the LE-supplemented endothelial cells (Figure 2). Lipid droplets were virtually undetected in non-supplemented endothelial cells. SO-supplemented cells consistently generated smaller lipid droplets compared to the much larger lipid droplets present in the OO- and FO-supplemented endothelial cells. Larger lipid droplets were consistent with the higher triglyceride levels in cells supplemented with OO (51%) or FO (61%) (Table 5a).

### Effect of lipid emulsion supplementation on HAEC apoptosis/necrosis

Endothelial cell viability was assessed after exposure of the cells to 0–0.5% v/v of LE. Cells were subsequently classified as viable, apoptotic, or necrotic (Table 6). OO supplementation of the endothelial cells improved cell viability. No statistically significant differences were observed within any cell classification in the SO-supplemented endothelial cells. FO supplementation of the cells decreased viability, demonstrating increases in both apoptosis and necrosis.

**Table 6:** **Effect of lipid emulsion supplementation on endothelial cell apoptosis/necrosis**

	**Apoptosis/necrosis detection (mean ± SD)**			
	**% (v/v)**	**Viable**	**Apoptotic**	**Necrotic**
Vehicle		82.9 ± 1.9	6.2 ± 1.8	10.9 ± 1.9
OO	0.5	86.8 ± 1.3*	6.0 ± 0.6	7.2 ± 1.0*
	0.1	87.7 ± 0.4*	5.9 ± 0.2	6.5 ± 0.3*
	0.05	84.7 ± 2.0	6.8 ± 1.0	8.5 ± 1.0
	0.025	84.5 ± 0.1	7.2 ± 0.8	7.9 ± 1.0
SO	0.5	81.6 ± 0.6	4.7 ± 1.2	13.8 ± 1.6
	0.1	85.6 ± 1.3	5.7 ± 0.9	8.7 ± 1.9
	0.05	84.1 ± 2.2	4.6 ± 0.5	11.3 ± 2.3
	0.025	82.7 ± 2.5	3.7 ± 0.9	12.9 ± 1.8
FO	0.5	64.2 ± 3.1*	12.9 ± 2.1*	22.9 ± 1.3*
	0.1	63.8 ± 2.0*	10.1 ± 3.3*	26.1 ± 4.6*
	0.05	76.8 ± 1.5*	6.9 ± 0.6	16.4 ± 1.4*
	0.025	79.9 ± 1.7	6.7 ± 0.7	13.4 ± 1.2

HAECs were supplemented with varying doses of lipid emulsions for 24 hours under standard tissue culture conditions. Cells were harvested and labeled with Annexin V FLUOS and propidium iodide as described in the methods. Results are expressed as the mean ± SD of three experiments. *Denotes statistical differences (p ≤ 0.05) within each cell classification compared to vehicle control.

### Effect of lipid emulsion supplementation on lipopolysaccharide-induced ICAM-1 surface expression

**Figure 3:**
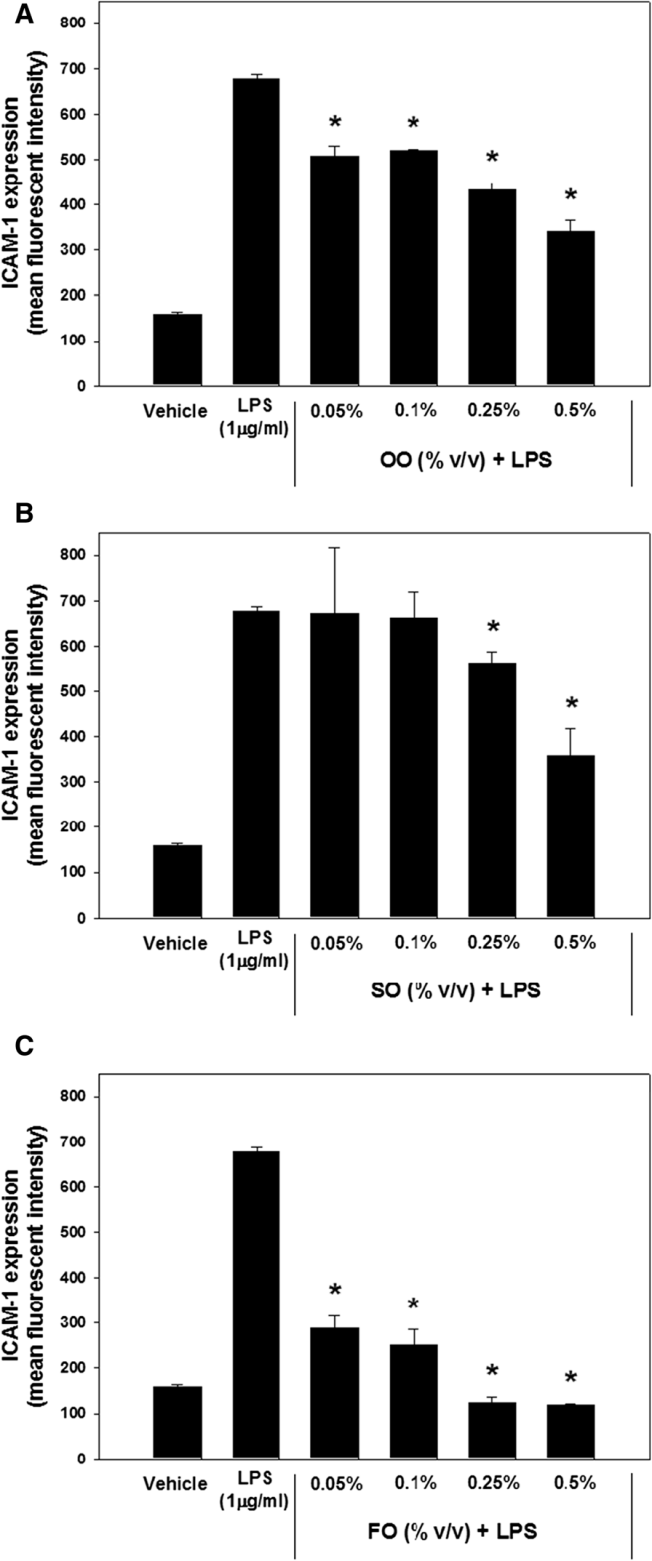
**Inhibitory effect of lipid emulsion supplementation on LPS-induced ICAM-1 surface expression.** Endothelial cells were supplemented with varying doses of OO **(A)**, SO **(B)**, or FO **(C)** for 24 hours under standard tissue culture conditions, followed by a 4-hour stimulation with or without LPS (1 μg/ml). Data are expressed as the mean ± SD of three determinations. A “*” denotes statistically significant inhibition from the LPS-stimulated positive control.

LPS-induced surface expression of ICAM-1 is utilized as a method to assess a proinflammatory response in endothelial cells; therefore, we used this method to determine if endothelial cells pretreated with LE were capable of modulating the LPS-induced inflammatory response. LPS-induced ICAM-1 expression increased nearly five-fold over levels in non-stimulated endothelial cells (Figure 3). Statistically significant decreases in ICAM-1 expression were observed in OO-supplemented cells, but the highest amount of OO (0.5% v/v) was only able to suppress the LPS-induced response by roughly one-half. In the SO- supplemented cells, ICAM-1 expression was decreased at the higher but not the lower doses of the emulsion. LPS-induced ICAM-1 expression was significantly inhibited following all doses of FO. Both the 0.25% and 0.5% FO emulsion doses completely inhibited ICAM-1 expression in the cells. To ensure that the LE were not directly interfering with the LPS stimulating effect, ICAM-1 expression levels were also quantified in cells that underwent two washes to remove residual LE prior to LPS stimulation, and results were compared with the unwashed cells. No significant differences existed between the repeatedly washed samples and the unwashed cells (data not shown).

### Effect of lipid emulsion supplementation on NF-kΒ phosphorylation

**Figure 4:**
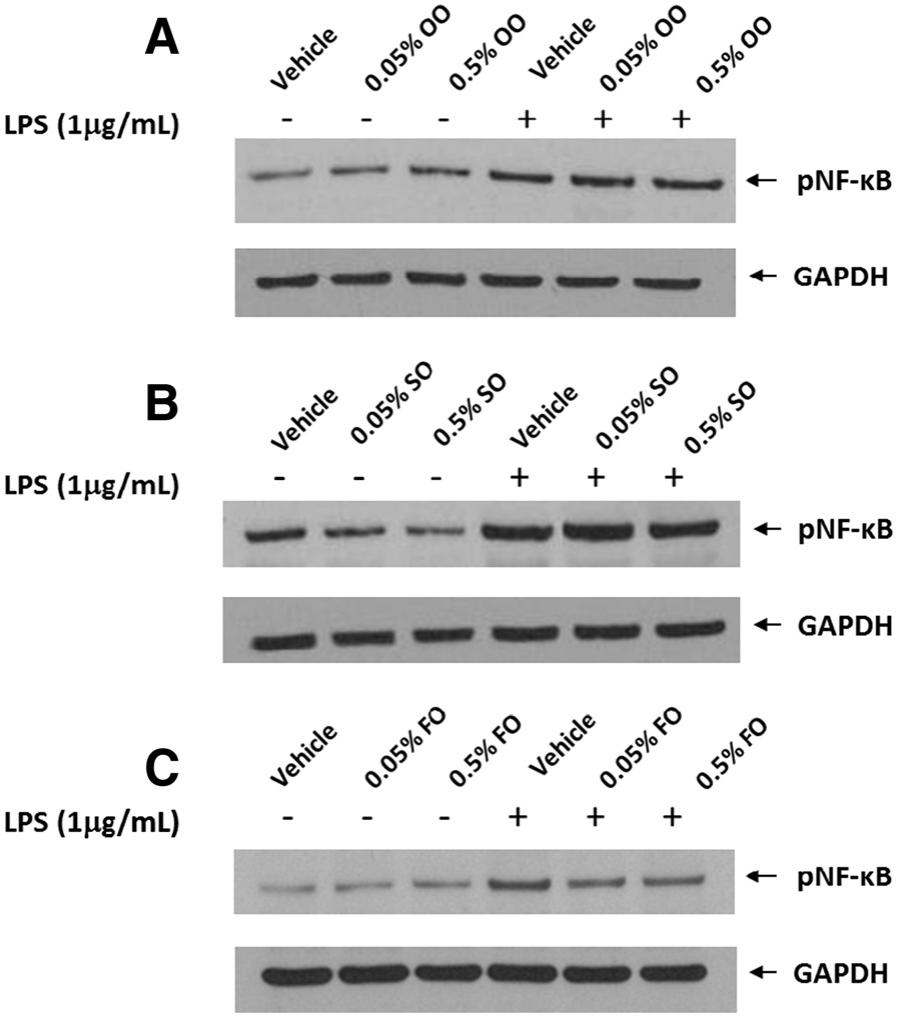
**Effect of lipid emulsion supplementation on phosphorylation of NF-κB.** Endothelial cells were pretreated with OO **(A)**, SO **(B)**, or FO **(C)** for 24 hours under standard tissue culture conditions, followed by a 4-hour stimulation with or without LPS (1 μg/ml). Cell lysates were electrophoretically separated in a 4-12% polyacrylamide gradient gel and transferred onto nitrocellulose membranes. A phospho-specific antibody to the p65 regulatory subunit of NF-κB assessed the relative activation state of the enzyme. Glyceraldehyde 3-phosphate dehydrogenase (GAPDH) served as the protein-loading control.

ICAM-1 expression is stimulated by activation (phosphorylation) of the transcription factor NF-κB. Thus, to determine if the anti-inflammatory properties (suppression of ICAM-1 expression) of the LEs were linked to suppression of NF-κB phosphorylation, we measured this molecule in both unstimulated and LPS-stimulated cells before and following exposure to the LE (0.05-0.5% v/v). In Figure 4A, LPS-stimulated endothelial cells demonstrated elevated expression of phosphorylated NF-κB regardless of whether cells were pretreated with OO, which was administered at levels previously shown to significantly inhibit ICAM-1 surface expression. OO supplementation did not appear to alter baseline NF-κB phosphorylation. SO supplementation partially lowered baseline NF-κB phosphorylation; however, the LPS-induced phosphorylation was not altered by the SO pretreatment (Figure 4B). FO supplementation did not affect baseline NF-κB phosphorylation. However, FO exposure decreased LPS-induced NF-κB phosphorylation (Figure 4C). Interestingly, all three LEs were capable of inhibiting ICAM-1 surface expression; however, only FO supplemented endothelial cells demonstrated decreased ICAM-1 expression and NF-κB phosphorylation.

## Discussion

Patients receiving parenteral nutrition frequently suffer from intravenous thrombosis and dysfunctional immune systems as a result of underlying disease (including pro-inflammatory insults), endothelial cell damage, disruption of the gastrointestinal-immune axis, malnutrition, and nutrient deficiencies/excesses. Loss of endothelial cell integrity (necrosis and apoptosis) predisposes to thrombosis by disrupting endothelial anticoagulant activities as well as contributing to the activation of coagulation. Dysregulation of immune and inflammatory responses during parenteral nutrition contributes to infections and/or tissue damage and organ failure.

Despite the importance of endothelial cells in the prevention of thrombosis and support of immune/inflammatory responses, few previous studies have evaluated the effects of LEs upon these endothelial cell functions. Thus, in this study we evaluated the effects of three major classes of commercial LEs. We were particularly interested in evaluating the pro-inflammatory versus anti-inflammatory potential of the three LEs upon endothelial cell integrity and function. Our results indicate that the n-6 predominant, n-3 predominant, and n-9 predominant LEs all suppress inflammatory endothelial cell activation. However, the dose–response effect indicates a difference in potency with the n-3 PUFA > n-9 MUFA > n-6 PUFA predominant LE. We found no evidence that any of the LEs increased the LPS-induced ICAM-1 response. Interestingly, we found that the fish oil emulsion increased both endothelial cell apoptosis and necrosis; the soybean oil emulsion had no effect upon these endothelial parameters, and the olive oil-predominant emulsion decreased necrosis without altering apoptosis.

LE effects upon endothelial cells primarily result from their uptake and incorporation into cell membranes and cytoplasmic compartments, where they modulate cell membrane structure-function, generation of cell signaling molecules (such as second messengers and transcription factors), and alter gene transcription. Our results indicate that all three LE were readily incorporated into endothelial cells in a dose-dependent manner (Tables 2, 3, 4 and Figure 1). The fatty acid profiles within the endothelial cells generally reflected the composition of the emulsions (Tables 1, 2, 3 and 4). Interestingly, we also found that the amount of fatty acid incorporated into endothelial cells varied among the different LEs. Significantly greater fatty acid was incorporated using the OO compared to the SO and FO emulsions (Figure 1). The FO emulsion was a 10% LE compared to the 20% LEs used for OO and SO. However, even when adjusted for the concentration of fatty acid administered, the OO emulsion was incorporated at higher levels than the other emulsions. When adjusted for fatty acid dose administered, the FO emulsion was incorporated at higher levels than the SO emulsion.

The phospholipid fraction within endothelial cells represents fatty acid content within cell surface and organelle membranes, while the triglyceride content represents cytoplasmic lipid or lipid within vesicles (such as pinocytotic vesicles or peroxisomes). Increases in fatty acids occurred in both the triglyceride and phospholipid fractions of the cell. Non-supplemented endothelial cells possessed a relatively small amount of triglycerides (4.5% of total fatty), whereas the percentages of triglyceride content were significantly elevated in OO (51.14%), SO (36.14%), and FO (60.6%) supplemented endothelial cells. Increased cellular lipid uptake from the LE was packaged and stored in the form of neutral lipids, namely triglycerides, as demonstrated by red oil O staining. Interestingly, SO was associated with numerous small droplets, while OO and FO were characterized by fewer but larger droplets. The larger droplets were consistent with higher cellular triglyceride content. From our previous study [8], we found that these intracellular lipid droplets disappear over time when the lipid emulsion is removed from the cells indicating that the lipid can be metabolized. The difference in speed of cellular uptake/incorporation, and the size and number of the droplets suggests that there is a difference in cellular lipid processing between the different emulsions. The mechanisms for this difference and potential physiologic effects requires further study. It is clear from these *in vitro* studies that fatty acid content of endothelial cells is modulated by the fatty acid content of the LE.

Apoptosis/necrosis of cells may play a role is endothelial dysfunction during acute (i.e. sepsis, vascular permeability, thrombosis) and chronic diseases (ie. atherosclerosis, nutrient and oxygen transport into tissues) [7,21,22]. Endothelial cell apoptosis may increase vascular thrombogenicity by an alteration of membrane phospholipid structure, loss of endothelial anti-coagulant proteins, enhanced binding and activation of coagulant factors, and/or exposure of the underlying procoagulant subcellular matrix [21,22]. Loss of endothelial nitric oxide production and endothelial nitric oxide synthase activity may also lead to a procoagulant state [6]. Thrombosis is an important complication associated with administration of parenteral nutrition and the effects of lipids upon thrombogenicity represents an important area for study.

Nonesterified free fatty acids have been reported to induce apoptosis in endothelial cells [5-9]. Free fatty acids may induce apoptosis through generation of cellular oxidants [6,7] and activation of NF-κB [5-9]. The apoptosis-inducing effects of free fatty acids differ among different fatty acids. Some investigators have reported induction of apoptosis with saturated free fatty acids but not unsaturated free fatty acids [5,6,8]. In addition, mixing unsaturated free fatty acids with saturated free fatty acids inhibited induction of apoptosis [5,6,8]. Thus, there appear to be interactions among different fatty acids, which can modulate their cellular actions. Lipid emulsions are emulsified complex mixtures of different fatty acids that are administered in the form of triglycerides and phospholipids. The effects of these complex fatty acid mixtures upon endothelial apoptosis are unknown and likely different from that of free fatty acids and simple mixtures of free fatty acids.

In this study, we assessed endothelial cell viability (including apoptosis and necrosis) following LE supplementation. Interestingly, the effects upon cell viability differed between the three LEs. OO-supplemented cells demonstrated an enhancement in cell viability with a decreased necrotic cell population. SO-supplemented cells had no significant alteration in cell viability. In contrast, FO- supplemented cells demonstrated a significant loss in cell viability with increased apoptosis and necrosis. This finding is even more significant when one considers that the FO emulsion (10% or 10 g/dl) was half the concentration of the OO and SO emulsions (20% or 20 g/dl). Importantly, fish oil increased apoptosis despite decreasing NF-κB activation, suggesting that this effect was not mediated directly by NF-κB. The mechanisms responsible for these effects upon endothelial cell viability require additional study but suggest that LEs modulate the apoptotic and cell necrotic pathways differently. In-vivo investigations of the effects of LEs upon endothelial cell viability are important since thrombosis is a major complication from use of parenteral nutrition.

Vascular endothelium regulates the transmigration of leukocytes into tissues following infection and/or tissue damage. Suppression of leukocyte transmigration into sites of infection may impair the ability to clear infections and heal wounds. On the other hand, exacerbation of transmigration may result in excessive inflammation and tissue damage. Leukocytes are recruited to sites of inflammation via leukocyte-endothelial interactions that are regulated through a series of adhesion molecules [11,12,23]. Expression of ICAM-1 and other adhesion molecules are increased by inflammatory stimuli (i.e. LPS, TNF-α, IL-1β) [24-26].

In this study, we assessed endothelial inflammatory activation by measuring endothelial expression of ICAM-1. We demonstrated that endothelial cell exposure to LPS significantly increased cell surface expression of ICAM-1. We found that exposure to the LEs resulted in different patterns and degrees of suppression of ICAM-1. All three LEs suppressed ICAM-1 in a dose-dependent manner. OO and SO were equally effective at suppressing LPS-stimulated ICAM-1 expression at the higher doses (0.5%); however, OO was more effective at the lower LE doses (0.05, 0.1, 0.25%). Maximal suppression for OO and SO was approximately 50%. The FO emulsion suppressed ICAM-1 expression in response to LPS at all doses studied (0.05-0.5%). Since the lipid concentration of FO was half that of OO or SO, the potency of ICAM-1 suppression was much greater than that of either OO or SO. In addition, FO at 0.25% and 0.5% completely suppressed the LPS-induced expression of ICAM-1. These results would indicate that FO has a greater anti-inflammatory effect upon endothelial cell ICAM-1 expression than the other LEs. However, it should be noted that all three emulsions demonstrated anti-inflammatory effects and none enhanced the ICAM-1 response. The suppression of ICAM-1 may be beneficial or detrimental, depending upon the clinical condition. ICAM-1 is an essential component of leukocyte transmigration into sites of inflammation and suppression of the response could impair the ability of the immune response to control infection or repair injured tissues. On the other hand, suppression might have benefit during an enhanced inflammatory response associated with tissue damage.

The suppressant effects of the FO emulsion (high in omega-3 PUFAs) upon endothelial cell activation are in agreement with studies using omega-3 free fatty acids (i.e. DHA and/or EPA) [23,27-35]. Overall, DHA and/or EPA have been shown to suppress leukocyte adhesion to activated endothelial cells [23,28-32,35], suppress expression of adhesion molecules [23,28-31,33-35] and reduce activation of NF-κB. SO-based emulsion has no suppressive effect on endothelial cell activation by LPS. In contrast, the OO-based emulsion suppressed ICAM-1 expression, which likely results from its complex mixture of different fatty acids.

Previous studies of the inhibitory effects of fish oil emulsions upon endothelial cell activation (using different models than in our study) are consistent with our results [36-38]. However, reports of the effects of soybean oil LEs upon endothelial activation are variable with reports of increased effects [36], no effect [37,38], or inhibitory effects [39]. Our study is the first report of suppression of ICAM-1 expression by an olive-oil predominate LE. Buenestado et al. [39] evaluated the effects of an olive-oil based and soybean oil-based LE upon LPS-induced leukocyte-endothelial interactions in-vivo in rat mesentery using intravital microscopy. Leukocyte adhesion and emigration were inhibited by soybean (consistent with our results) but unaffected by the olive oil-based LE. ICAM-1 expression was not assessed. Consistent with our findings using an olive-oil predominate LE, olive oil supplemented oral diets have been shown to decrease expression of ICAM-1 by human peripheral blood mononuclear cells in healthy middle-aged men [40].

Previous studies have demonstrated that LPS, proinflammatory cytokines (TNFα, IL-1β), and fatty acids mediate endothelial activation (expression of adhesion molecules such as ICAM-1) via the transcription factor NF-κB [8,14,24-26,31,41,42] Activation of NF-κB occurs via two major routes [43,44]. In the inactive state, NF-κB is found in the cytoplasm bound to IkBα, which prevents it from entering the nucleus. In response to inflammatory stimuli, IκB kinase (IKK) is activated and phosphorylates IkBα. Phosphorylated IκBα is subsequently degraded, allowing for translocation of NF-κB complexes into the nucleus [45]. However, IKK phosphorylation of IkBα alone is not sufficient for NF-κB to initiate gene transcription. Optimal induction of NF-κB target genes also requires phosphorylation of NF-κB proteins [43,44]. Phosphorylation enhances NF-κB interactions with co-activator proteins and gene promoter regions [43]. Thus, measurement of phosphorylated NF-κB serves as an indicator of NF-κB activation. LPS-induced phosphorylation of NF-κB can lead to the transcription and subsequent translation of pro-inflammatory molecules, including ICAM-1 protein expression.

We measured NF-κB phosphorylation to determine if it represented a novel molecular mechanism for the observed effects of the LEs upon ICAM-1 expression. This is the first study that has evaluated NF-κB phosphorylation by LEs. Neither OO nor SO pretreatment altered the LPS-induced NF-κB phosphorylation; however, FO-supplemented endothelial cells exhibited diminished levels of NF-κB phosphorylation. These data suggest that FO supplementation decreases ICAM-1 expression wholly or partially through suppression of NF-κB activation. The suppression of ICAM-1 by OO and SO do not appear to involve suppression of NF-κB phosphorylation. For these emulsions, we speculate that the LE may alter endothelial cell membrane composition and prevent ICAM-1 incorporation into the cell membranes. Other possible mechanisms for suppression of ICAM-1 expression include interference with co-inducers or co-factors that interact with NF-κB to promote binding and transcription of the ICAM-1 gene, altered binding of NF-κB to the ICAM-1 promoter, induction of ICAM-1 inhibitor proteins, and interference with other transcription factors that regulate ICAM-1 expression [25]. Further studies are required to investigate these and other mechanisms for the diminished cell surface expression of ICAM-1 with LEs.

EPA and DHA, consistent with our findings using the fish oil-based LE, EPA and DHA have been demonstrated to inhibit endothelial adhesion molecule expression and leukocyte-endothelial adhesion and transmigration by inhibiting NF-κB activity [28,29,31,46]. On the other hand, linoleic acid has been reported to activate NF-κB and induce NF-κB-dependent transcription in endothelial cells [47-49]. However, these results were obtained with the single fatty acid, linoleic acid, and not the complex lipid emulsion enriched in linoleic acid (i.e. soybean oil emulsion). Oleic acid and EPA have been shown to inhibit NF-κB activation induced by saturated fatty acids in endothelial cells [6,8], indicating that one fatty acid may be able to inhibit the effects of other fatty acids. Our results using a complex lipid emulsion that contains high levels of linoleic acid along with other fatty acids indicates that soybean oil-based LE inhibits endothelial cell expression of ICAM-1 and has no effect on NF-κB activation through phosphorylation. Thus, complex fatty acid lipid mixtures act differently from purified single fatty acids.

The doses of triglycerides (from the LEs) used in this study include those normally found in the blood during fasting, the postprandial state, and in patients receiving parenteral nutrition with LE. Normal fasting serum triglyceride levels are less than 160 mg/dl [50-52]. Cohen et al. [51] reported that mean postprandial serum triglyceride levels varied from 140–210 mg/dl in normal volunteers (fat intake 40–80 grams) while Cohn et al. [52] reported mean values of 240 mg/dl (females) and 320 mg/dl (males) following 1 g/kg of fat intake. In a review of serum triglyceride levels in patients receiving LE as a component of parenteral nutrition, 7.6% had triglyceride levels greater than 400 mg/dl [53]. The mean level in these hypertriglyceridemic patients was 486.8 mg/dl; values improved when the LE was discontinued (274 mg/dl). Triglyceride levels in neonates receiving parenteral nutrition ranged from 20–536 mg/dl [54]. Others [55,56] reported mean triglyceride levels of approximately 200 mg/dl in patients receiving parenteral nutrition. Llop et al. [55] reported that 26.2% of surgical parenteral nutrition patients had triglyceride levels greater than 265 mg/dl. Levels of triglycerides may approach 500 mg/dl (based upon the mean + 2.5 SD) [55]. Most parenteral nutrition guidelines recommend that serum triglyceride levels be kept < 400 mg/dl. The dose of triglyceride used in this study for the cell viability, ICAM-1 modulation, and NF-κB phosphorylation experiments ranged from 0.025% to 0.5% (5–100 mg/dl for 20% LE; 2.5-50 mg/dl for the 10% LE). These values range from low to normal triglyceride levels. We used 0.1-10% LEs for the incorporation studies so as to span the entire physiologic range of triglycerides from very low levels (0.1%; 10–20 mg/dl), normal fasting and postprandial levels (1-2%; 100–400 mg/dl), to elevated levels (5-10%; 500–2000 mg/dl). Importantly, the endothelial cell viability and activation studies used triglyceride concentrations that were within normal serum ranges. However, *in vitro* experimentation has limitations and may significantly differ from *in vivo* cellular uptake of the lipid emulsion components. Additionally, other physiological factors may affect the inflammatory status on the endothelial cells *in vivo*. Furthermore, *in vivo* experimentation is necessary to determine if our *in vitro* observations translate into an *in vivo* model.

## Conclusions

This study demonstrates that commercial LEs composed of different oils produce different effects upon endothelial cell functions that include fatty acid uptake and incorporation, integrity, and inflammatory activation. Fatty acid incorporation into the cells demonstrated different cellular localization (i.e. phospholipid vs triglyceride compartments). The OO emulsion improved endothelial cell viability while the FO emulsion decreased cell viability (the SO emulsion had no effect on viability). Contrary to our initial hypothesis, we found that the n-3 PUFA-, n-6 PUFA-, and n-9 MUFA-predominant LEs all suppressed endothelial cell expression of ICAM-1 (an indicator of endothelial cell inflammatory response); however, the omega-3 PUFA emulsion was much more potent than the other emulsions. In addition, the n-3 PUFA-predominant LE inhibited activation (phosphorylation) of NF-κB, while the soybean and olive/soybean LEs did not alter NF-κB phosphorylation. These results suggest that n-3 PUFA-predominant LE inhibits endothelial adhesion molecule expression by a mechanism that differs from that of n-6 PUFA- or n-9 MUFA-predominant LEs. Future studies are required to determine whether the observed *in vitro* endothelial effects are predictive of *in vivo* effects, and whether different mechanisms of ICAM-1 inhibition have implications for disease development and progression.

## Abbreviations

FO: Fish oil
HAECs: Human aortic endothelial cells
LE(s): Lipid emulsion(s)
LPS: Lipopolysaccharide
ICAM-1: Intercellular adhesion molecule-1
NF-κB: Nuclear factor kappa B
OO: Olive oil
SO: Soybean oil
IL-1β: Interleukin-1 beta
PUFA: Polyunsaturated fatty acid
MUFA: Monounsaturated fatty acid
PBS: Phosphate buffered saline
EBM-2: Endothelial basal medium-2
EGM-2MV: Endothelial growth medium-2 microvascular
RIPA: Radioimmunoprecipitation assay
TBST: Tris buffered saline tween 20
DHA: Docosahexaenoic acid
EPA: Eicosapentaenoic acid
PE: Phycoerythrin
PL: Phospholipid
TG: Triglyceride
FBS: Fetal bovine serum
GAPDH: Glyceraldehyde 3-phosphate dehydrogenase
ECL: Enhanced chemiluminescence
